# Supplementary material for: Resolving the full spectrum of human genome variation using Linked-Reads
Source: Genome Res. 2019 Apr;29(4):635–45. doi: 10.1101/gr.234443.118 (PMC6442396; doi:10.1101/gr.234443.118)
Supplement: Supplemental Material [file supp_gr.234443.118_Supplemental_longranger-2.2.zip › longranger-2.2.tar.bz2!/lr2.2-public/lib/stan/cmdstan/stan/doc/api/html/classstan_1_1optimization_1_1_l_b_f_g_s_update.html]

Stan: stan::optimization::LBFGSUpdate< Scalar, DimAtCompile > Class Template Reference


|  |  |
| --- | --- |
|  | Stan  2.5.0  probability, sampling & optimization |


- Main Page
- Related Pages
- Namespaces
- Classes
- Files

- Class List
- Class Index
- Class Hierarchy
- Class Members

All Classes Namespaces Files Functions Variables Typedefs Enumerations Enumerator Friends Macros Pages

- stan
- optimization
- LBFGSUpdate

Public Types |
Public Member Functions |
Protected Attributes |
List of all members

stan::optimization::LBFGSUpdate< Scalar, DimAtCompile > Class Template Reference

Implement a limited memory version of the BFGS update.
More...

`#include <lbfgs_update.hpp>`

|  |  |
| --- | --- |
| Public Types | |
| typedef Eigen::Matrix< Scalar,   DimAtCompile, 1 > | VectorT |
|  | |
| typedef Eigen::Matrix< Scalar,   DimAtCompile, DimAtCompile > | HessianT |
|  | |
| typedef boost::tuple< Scalar,   VectorT, VectorT > | UpdateT |
|  | |

|  |  |
| --- | --- |
| Public Member Functions | |
|  | LBFGSUpdate (size\_t L=5) |
|  | |
| void | set\_history\_size (size\_t L) |
|  | Set the number of inverse Hessian updates to keep. More... |
|  | |
| Scalar | update (const VectorT &yk, const VectorT &sk, bool reset=false) |
|  | Add a new set of update vectors to the history. More... |
|  | |
| void | search\_direction (VectorT &pk, const VectorT &gk) const |
|  | Compute the search direction based on the current (inverse) Hessian approximation and given gradient. More... |
|  | |

|  |  |
| --- | --- |
| Protected Attributes | |
| boost::circular\_buffer< UpdateT > | \_buf |
|  | |
| Scalar | \_gammak |
|  | |

## Detailed Description

### template<typename Scalar = double, int DimAtCompile = Eigen::Dynamic> class stan::optimization::LBFGSUpdate< Scalar, DimAtCompile >

Implement a limited memory version of the BFGS update.

This class maintains a circular buffer of inverse Hessian updates which can be applied to compute the search direction.

Definition at line 20 of file lbfgs\_update.hpp.

## Member Typedef Documentation

template<typename Scalar = double, int DimAtCompile = Eigen::Dynamic>

typedef Eigen::Matrix<Scalar,DimAtCompile,DimAtCompile> stan::optimization::LBFGSUpdate< Scalar, DimAtCompile >::HessianT

Definition at line 23 of file lbfgs\_update.hpp.

template<typename Scalar = double, int DimAtCompile = Eigen::Dynamic>

typedef boost::tuple<Scalar,VectorT,VectorT> stan::optimization::LBFGSUpdate< Scalar, DimAtCompile >::UpdateT

Definition at line 24 of file lbfgs\_update.hpp.

template<typename Scalar = double, int DimAtCompile = Eigen::Dynamic>

typedef Eigen::Matrix<Scalar,DimAtCompile,1> stan::optimization::LBFGSUpdate< Scalar, DimAtCompile >::VectorT

Definition at line 22 of file lbfgs\_update.hpp.

## Constructor & Destructor Documentation

template<typename Scalar = double, int DimAtCompile = Eigen::Dynamic>

|  |  |  |  |  |  |
| --- | --- | --- | --- | --- | --- |
| stan::optimization::LBFGSUpdate< Scalar, DimAtCompile >::LBFGSUpdate | ( | size\_t | *L* = `5` | ) |  |

inline

Definition at line 26 of file lbfgs\_update.hpp.

## Member Function Documentation

template<typename Scalar = double, int DimAtCompile = Eigen::Dynamic>

|  |  |  |  |
| --- | --- | --- | --- |
| void stan::optimization::LBFGSUpdate< Scalar, DimAtCompile >::search\_direction | ( | VectorT & | *pk*, |
|  |  | const VectorT & | *gk* |
|  | ) |  | const |

inline

Compute the search direction based on the current (inverse) Hessian approximation and given gradient.

Parameters
:   |  |  |  |
    | --- | --- | --- |
    | [out] | pk | The negative product of the inverse Hessian and gradient direction gk. |
    | [in] | gk | Gradient direction. |

Definition at line 78 of file lbfgs\_update.hpp.

template<typename Scalar = double, int DimAtCompile = Eigen::Dynamic>

|  |  |  |  |  |  |
| --- | --- | --- | --- | --- | --- |
| void stan::optimization::LBFGSUpdate< Scalar, DimAtCompile >::set\_history\_size | ( | size\_t | *L* | ) |  |

inline

Set the number of inverse Hessian updates to keep.

Parameters
:   |  |  |
    | --- | --- |
    | L | New size of buffer. |

Definition at line 33 of file lbfgs\_update.hpp.

template<typename Scalar = double, int DimAtCompile = Eigen::Dynamic>

|  |  |  |  |
| --- | --- | --- | --- |
| Scalar stan::optimization::LBFGSUpdate< Scalar, DimAtCompile >::update | ( | const VectorT & | *yk*, |
|  |  | const VectorT & | *sk*, |
|  |  | bool | *reset* = `false` |
|  | ) |  |  |

inline

Add a new set of update vectors to the history.

Parameters
:   |  |  |
    | --- | --- |
    | yk | Difference between the current and previous gradient vector. |
    | sk | Difference between the current and previous state vector. |
    | reset | Whether to reset the approximation, forgetting about previous values. |

Returns
:   In the case of a reset, returns the optimal scaling of the initial Hessian approximation which is useful for predicting step-sizes.

Definition at line 48 of file lbfgs\_update.hpp.

## Member Data Documentation

template<typename Scalar = double, int DimAtCompile = Eigen::Dynamic>

boost::circular\_buffer<UpdateT> stan::optimization::LBFGSUpdate< Scalar, DimAtCompile >::\_buf

protected

Definition at line 113 of file lbfgs\_update.hpp.

template<typename Scalar = double, int DimAtCompile = Eigen::Dynamic>

Scalar stan::optimization::LBFGSUpdate< Scalar, DimAtCompile >::\_gammak

protected

Definition at line 114 of file lbfgs\_update.hpp.

---

The documentation for this class was generated from the following file:

- src/stan/optimization/lbfgs\_update.hpp


---

[ Stan Home Page ]

*© 2011–2014,
Stan Development Team.*
